# Association Between Per- and Polyfluoroalkyl Substances and All-Cause Mortality in Diabetic Patients: Insights from a National Cohort Study and Toxicogenomic Analysis

**DOI:** 10.3390/toxics13030168

**Published:** 2025-02-27

**Authors:** Zhengxiao Wei, Jinyu Chen, Xue Mei, Yi Yu

**Affiliations:** 1 Department of Clinical Laboratory, Public Health Clinical Center of Chengdu, Chengdu 610061, China; weizhengxiao@outlook.com; 2 Department of Tuberculosis, Public Health Clinical Center of Chengdu, Chengdu 610061, China; bell_jychen@outlook.com; 3 Department of Infectious Diseases, Public Health Clinical Center of Chengdu, Chengdu 610061, China; meixuexiaoxie@163.com; 4 Department of Infectious Diseases, The Second Hospital of Lanzhou University, Lanzhou 730030, China

**Keywords:** per- and polyfluoroalkyl substances, perfluorooctane sulfonate, diabetes mellitus, all-cause mortality

## Abstract

Per- and polyfluoroalkyl substances (PFAS) are a group of environmental contaminants associated with various health risks; however, their relationship with all-cause mortality in individuals with diabetes remains unclear. A total of 1256 participants from the National Health and Nutrition Examination Survey (NHANES) were included to explore the association between seven PFAS compounds and all-cause mortality in diabetic patients. Preliminary logistic regression identified three PFAS compounds (perfluorooctanoic acid [PFOA], perfluorooctane sulfonic acid [PFOS], and 2-(N-methyl-PFOSA) acetate acid [MPAH]) as significantly associated with mortality in the diabetic population. The optimal cut-off values for PFOS, PFOA, and MPAH were determined using the X-tile algorithm, and participants were categorized into high- and low-exposure groups. Kaplan–Meier survival curves and multivariable Cox proportional hazards regression models were used to assess the relationship between PFAS levels and mortality risk. The results showed that high levels of PFOS were significantly associated with increased all-cause mortality risk in diabetic patients (hazard ratio [HR]: 1.55, 95% confidence interval [CI]: 1.06–2.29), while PFOA and MPAH showed no significant associations. To explore mechanisms underlying the PFOS–mortality link, toxicogenomic analysis identified 95 overlapping genes associated with PFOS exposure and diabetes-related mortality using the Comparative Toxicogenomics Database (CTD) and GeneCards. Functional enrichment analysis revealed key biological processes, such as *glucose homeostasis* and *response to peptide hormone*, with pathways including the *longevity regulating pathway*, *apoptosis*, and *p53 signaling pathway*. Protein–protein interaction network analysis identified 10 hub genes, and PFOS was found to upregulate or downregulate their mRNA expression, protein activity, or protein expression, with notable effects on mRNA levels. These findings suggest that PFOS exposure contributes to increased mortality risk in diabetic patients through pathways related to glucose metabolism, apoptosis, and cellular signaling. Our study provides new insights into the association between PFAS and all-cause mortality in diabetes, highlighting the need for large-scale cohort studies and further in vivo and in vitro experiments to validate these findings.

## 1. Introduction

Diabetes mellitus (DM) is a progressively worsening metabolic disorder of global concern, imposing substantial economic burdens on both patients and society. The International Diabetes Federation (IDF) reports a continuous rise in diabetes prevalence, with affected individuals increasing from 100 million in 1980 to over 500 million in 2021 and projections indicating a rise to 548 million by 2045 [1]. This condition not only diminishes the quality of life for patients but also significantly elevates the risks of cardiovascular disease, kidney disease, and all-cause mortality. Individuals with diabetes face a markedly higher risk of myocardial infarction, stroke, and other cardiovascular events compared to non-diabetic populations [2]. Chronic hyperglycemia damages glomerular structures, leading to diabetic nephropathy, and the survival rate of patients with diabetes-related chronic kidney disease (CKD) is significantly lower than that of non-diabetic individuals [3]. Moreover, all-cause mortality among diabetic patients, particularly due to cardiovascular diseases, renal failure, and infections, is substantially higher than in the general population [4].

Recent studies have focused on the potential connection between environmental pollutants, especially per- and polyfluoroalkyl substances (PFAS), and metabolic disease development and progression [5]. PFAS are synthetic compounds known for their remarkable chemical stability [6] and are extensively utilized in industrial and consumer products such as waterproof coatings, firefighting foams, food packaging, and electronic devices [7]. While their unique physicochemical properties have provided considerable commercial value, the persistence of PFAS in the environment and their bioaccumulative nature have raised significant concerns for global environmental pollution and public health [8]. PFAS compounds, such as perfluorooctane sulfonate (PFOS) and perfluorooctanoic acid (PFOA), are highly resistant to degradation, resulting in their prolonged presence in water, soil, air, and biological systems and their accumulation through food web [9]. PFAS have been detected in drinking water worldwide, with over 200 million people in the United States exposed to PFAS in their drinking water [10]. Epidemiological and experimental evidence suggests that exposure to PFAS may be associated with a range of chronic diseases, particularly metabolic disorders like diabetes [11]. Research indicates that PFAS may disrupt insulin signaling pathways [12], contributing to insulin resistance [13], a key factor in the development of type 2 diabetes. Additionally, PFAS may induce chronic inflammation [14], impair pancreatic β-cell function [15], and promote lipid metabolism disorders, further exacerbating metabolic disturbances [16]. While existing research indicates a significant link between PFAS exposure and the development of diabetes and metabolic disorders, the connection between PFAS and all-cause mortality in diabetic individuals is not well studied.

To address this knowledge gap, the present study conducted a prospective analysis based on follow-up data from the U.S. National Health and Nutrition Examination Survey (NHANES) from 1999 to 2018, focusing on individuals with diabetes. The study hypothesized that a higher concentration of per- and polyfluoroalkyl substances (PFAS) in the body could be associated with higher all-cause mortality in diabetic patients. To test this hypothesis, we developed four multivariable-adjusted models, accounting for potential covariates such as age, body mass index, race, sex, smoking status, and socioeconomic status. These models systematically evaluated the potential association between the concentrations of seven PFAS compounds and all-cause mortality in diabetic individuals. Additionally, we utilized the Comparative Toxicogenomics Database (CTD) to identify potential biomolecular mechanisms and intervention targets related to the specified target PFAS that are associated with diabetes mortality. The findings of this study aim to enhance the understanding of potential health risks associated with PFAS exposure in diabetic patients, which may provide new insights and strategies for future research.

## 2. Methods

### 2.1. Population-Based Cohort Study Analysis

#### 2.1.1. Research Population

**Figure 1:**
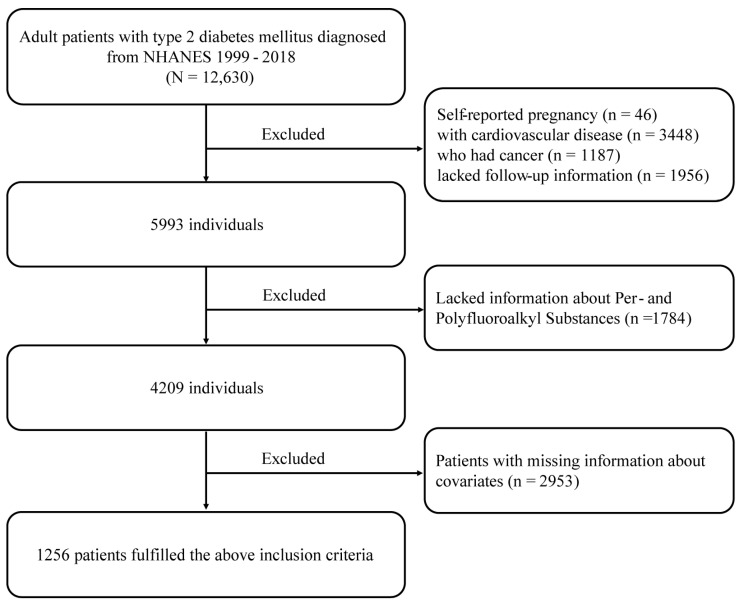
Flowchart of the study population.

The study population was sourced from the NHANES conducted in the U.S. from 1999 to 2018, with follow-up until 31 December 2019. The National Center for Health Statistics (NCHS) conducts NHANES, which comprises interviews, physical examinations at home or in mobile centers, and laboratory tests. The survey utilizes a complex, stratified, multistage probability sampling design implemented biennially. Comprehensive details on sampling methods and data collection procedures can be found on the NHANES website (http://www.cdc.gov/nchs/nhanes.htm, accessed on 1 September 2024). The study received approval from the Institutional Review Board of the Centers for Disease Control and Prevention (CDC), and all participants gave written informed consent. Participants were identified as having diabetes if they (1) self-reported a diabetes diagnosis; (2) reported using insulin or antidiabetic medications; or (3) exhibited HbA1c levels ≥6.5% or fasting plasma glucose levels ≥7.0 mmol/L [17]. Among the 101,316 participants in the NHANES survey, we initially selected individuals aged ≥18 years with type 2 diabetes (T2D), resulting in a sample of 12,630 diabetic subjects. After excluding participants who were self-reported pregnant (*n* = 46), had cardiovascular disease (CVD) (*n* = 3448), had cancer (*n* = 1187), or lacked follow-up data (*n* = 1956), 5993 adult diabetic patients remained. Further exclusions were made for missing PFAS data (*n* = 1784) and missing covariate data (*n* = 2958). Ultimately, 1256 diabetic patients were included in this study (Figure 1).

#### 2.1.2. Measurement of Serum PFAS Compounds

In the NHANES data, serum samples were sent to the Centers for Disease Control and Prevention (CDC) for examination, and samples were stored at −20 °C before being transported to the laboratory for testing, and serum PFAS concentrations were measured using solid-phase extraction coupled with high-performance liquid chromatography–isotope dilution–tandem mass spectrometry, following the detailed description of the analytical methodology previously published. Serum PFAS concentrations were assessed in a random subsample representing about one-third of the total NHANES population during each cycle. In cycles from 1999 to 2012, serum measurements were conducted for seven PFAS compounds: PFOS, PFOA, perfluorononanoic acid (PFNA), perfluorohexanesulfonic acid (PFHxS), perfluoroundecanoic acid (PFUS), fluorodecanoic acid (PFDE), and 2-(N-methyl-PFOSA) acetate acid (MPAH). Typically, total concentrations of PFOS and PFOA are measured. Between 2013 and 2018, linear and branched isomers of specific PFAS compounds, such as linear and branched PFOA isomers as well as linear and monomethyl-branched PFOS isomers, were measured using a consistent method. To ensure consistency, we determined the total concentrations of PFOS and PFOA for the 2013–2018 survey cycles by aggregating the levels of their linear and branched isomers [18,19]. For concentrations below the detection threshold, The values were substituted with the lower limit of detection (LLOD) divided by square root of 2 (LLOD/sqrt(2)). PFAS levels were measured at baseline. Follow-up for health outcomes commenced from the baseline PFAS measurement, and any exclusions were based on participants’ health status at that time. For comprehensive details on quality control procedures, refer to the NHANES Laboratory Procedures Manual.

#### 2.1.3. Definition of Outcome

The main focus of this study was all-cause mortality. Briefly, the NCHS linked NHANES 1999–2018 participants to potential causes of death in the National Death Index using probabilistic matching criteria based on identifiers such as Social Security numbers and birth dates. Participants were followed up until 31 December 2019. If no match was found in the National Death Index, the participant was presumed to be alive as of that date. For NHANES 1999–2014, the publicly available linked mortality files included nine specific categories of cause of death. However, for NHANES 2015–2018, due to the shorter follow-up period and smaller sample sizes for certain specific causes of death, the publicly available linked mortality files included only two specific categories: heart disease and cancer.

#### 2.1.4. The Use of Covariates

Demographic data were collected via self-reported questionnaires, encompassing sex (male/female), age (18–59/≥60), race (white/black/other), educational attainment (less than high school/high school/college or higher), and poverty income ratios (PIR) (<1.3, 1.3–3.5, >3.5). The PIR is the ratio of household income to the poverty standard. Health technicians assessed participants’ height and weight, classifying adults with a body mass index (BMI) (<18, 18–25, >25). The Healthy Eating Index 2015 (HEI-2015) score, reflecting diet quality based on 13 food and nutrient components, was calculated using the average intake from two dietary recalls, with higher scores indicating superior diet quality. For an in-depth explanation of the HEI-2015, refer to source [20]. In addition, we considered drinking status (never, former, moderate, mild, and heavy), smoking status (never, former, and now), total cholesterol content (mg/mL), and whether or not the participant was taking anti-diabetic medication.

#### 2.1.5. Statistical Analysis

Analyses were conducted using R software (version 4.3.1). Continuous variables are expressed as mean (SD) or median (interquartile range, IQR), while categorical variables are reported as absolute counts with frequencies (percentages, %). Following NHANES statistical guidelines, we accounted for the sampling design and weights of each survey cycle in the analytical model using the “svyglm” function in R. All analyses were adjusted for predefined covariates by including them in the models. The associations between seven PFAS compounds and all-cause mortality among diabetic patients were evaluated using univariate and multivariable logistic regression analyses. For each compound, four statistical models were developed. The crude model was not adjusted. Model 1 was adjusted for variables including age, sex, race, education, and the PIR. Model 2 was additionally adjusted for BMI, smoking, alcohol consumption, and the HEI-2015. Model 3 additionally accounted for hypertension, total cholesterol levels, and medication use. Results are presented as odds ratios (ORs) with 95% confidence intervals (CIs). Multivariable logistic regression identified PFOA, PFOS, and MPAH as significantly associated with all-cause mortality, warranting further analysis.

The diagnostic value of these compounds for predicting mortality risk was evaluated using receiver operating characteristic (ROC) curve analysis, with the area under the curve (AUC) calculated to measure predictive efficacy. The optimal cut-off concentrations for PFOA, PFOS, and MPAH were determined using the X-tile software (version 3.6.1), a statistical tool designed to identify the optimal thresholds for continuous variables by maximizing their association with outcomes of interest, such as survival time, risk distribution, or disease status, based on chi-square values [21]. Using these cut-off points, participants were classified into high-level and low-level exposure groups. Kaplan–Meier survival analysis compared survival curves between high- and low-exposure groups, with the log-rank test assessing statistical significance. A Cox proportional hazards regression model was used to further analyze compounds significantly associated with all-cause mortality in diabetic patients. The Cox model estimated hazard ratios (HRs) with 95% confidence intervals (CIs) to assess the independent impact of PFAS exposure on mortality risk, adjusting for covariates such as age, sex, BMI, PIR, education level, race, smoking, alcohol consumption, HEI-2015, hypertension, total cholesterol, and medication use. Finally, to evaluate the robustness of the association results, two sensitivity analyses were performed. First, we excluded diabetic patients who died within one year of enrollment. Second, we constructed a directed acyclic graph (DAG) to identify confounders that were associated with both exposure and outcome, and based on this, we determined the minimal set of adjustment variables. We then repeated the analysis using a Cox proportional hazards model.

### 2.2. Toxicogenomic Analysis

#### 2.2.1. Identification of Potential Target Genes for PFAS Using Databases

The Comparative Toxicogenomics Database (CTD, https://ctdbase.org/, accessed on 25 September 2024) is a publicly available, manually curated resource that includes over 16,300 chemicals and their interactions with genes and proteins. The database also integrates information on the relationships between genes and diseases, chemicals and diseases, and chemical exposures and phenotypes, providing valuable insights into the biological effects of environmental exposures [22]. In this study, we initially identified diabetes-related genes associated with PFAS compounds from the CTD database. Subsequently, we filtered genes related to “death due to diabetes” using the GeneCards database (version 3), a comprehensive platform providing detailed gene information, including functional predictions and expression patterns, to enhance understanding of disease mechanisms [23]. Finally, we identified the overlapping genes between PFAS-related diabetes genes and “diabetes death” genes, visualizing the intersection using Venn diagrams.

#### 2.2.2. Gene Ontology (GO) and Kyoto Encyclopedia of Genes and Genomes (KEGG) Pathway Enrichment Analysis

GO analysis provides annotations for gene products from three perspectives: function, pathways, and cellular localization. These are categorized into three main ontologies: biological process (BP), which describes the biological objectives or processes in which the gene products are involved; cellular component (CC), which defines the subcellular locations where these gene products function; and molecular function (MF), which details the specific biochemical activities or actions performed by the gene products. KEGG analysis integrates genomic, chemical, and system functional information, including gene pathway data. These two analyses offer a more comprehensive understanding of gene functions and the biological processes in which they participate. We performed GO and KEGG pathway enrichment analysis using the R package “clusterProfiler”, applying a false-discovery rate (FDR) threshold of *p* < 0.05.

#### 2.2.3. Construction of PPI Network and Identification of Hub Genes

To explore the interactions among the target genes, we constructed a protein–protein interaction (PPI) network using STRING (parameters: Organisms: *Homo sapiens*, Network Type: Full STRING Network, Required score: Highest confidence (0.700), FDR stringency: Medium (5%)). Subsequently, we employed the CytoHubba Cytoscape plugin (https://apps.cytoscape.org/apps/cytohubbawa, (version 3.8.0)) to identify the top 10 hub genes based on the maximum clique centrality (MCC) method, using default settings for the parameters.

#### 2.2.4. Chemical–Gene Interaction Patterns

We used the CTD database to examine the interaction patterns between the identified hub genes and PFAS compounds. This analysis focused exclusively on direct interactions between individual chemical substances and single genes, excluding complex interactions involving multiple genes or chemicals.

## 3. Results

### 3.1. Baseline Characteristics

This study included 1256 diabetic participants from the NHANES survey. These participants had comprehensive data on PFAS compound concentrations and associated covariates. Table 1 summarizes the baseline characteristics of these participants. Overall, 50.95% of the participants were female, and more than half were non-Hispanic white. Over a median follow-up period of 98 months (IQR: 57–142), 212 deaths were observed among the diabetic participants. Compared to survivors, decedents were characterized by being older, more likely to belong to the middle-income group, predominantly non-Hispanic white, with lower education levels (high school or less), and a higher proportion of current or former smokers and drinkers. Additionally, the deceased had a relatively lower BMI and a higher prevalence of hypertension. Analysis of the seven PFAS compounds revealed that the concentrations of three compounds (PFOS, PFOA, and MPAH) were substantially elevated in the mortality group (*p* < 0.05). In contrast, the concentrations of the other four PFAS compounds (PFNA, PFHxS, PFDE, and PFUA) indicated no significant differences across the groups (*p* > 0.05).

### 3.2. Logistic Regression Analysis of PFAS Compounds and Death in the DM Population

As shown in Table 2, univariate logistic regression analysis revealed that the concentrations of three PFAS compounds (PFOS, PFOA, and MPAH) were significantly positively associated with mortality in diabetic patients. Specifically, a 1-unit rise in PFOA (ng/mL), PFOS (ng/mL), and MPAH (ng/mL) concentrations resulted in a mortality risk in diabetic patients that was increased by 9%, 3%, and 151%, respectively. Further, we constructed three multivariable-adjusted logistic regression models to control for covariates such as age, sex, BMI, PIR, education level, race, smoking, alcohol consumption, HEI-2015, hypertension, total cholesterol, and medication use. After adjustment, the concentrations of PFOS, PFOA, and MPAH remained significantly associated with mortality, and the results from the multivariable-adjusted models were consistent with those from the unadjusted models. This suggests a strong positive correlation between the concentrations of these compounds and mortality in diabetic patients. The results indicated that a 1-unit increase in PFOS concentration was associated with a 3% rise in mortality (95% CI: 1.01–1.04), a 1-unit increase in PFOA concentration corresponded to an 11% rise (95% CI: 1.03–1.19), and a 1-unit increase in MPAH concentration led to a 97% rise (95% CI: 1.39–2.81).

### 3.3. Assessing the Predictive Value of PFAS Compounds for All-Cause Mortality in DM Patients

To further evaluate the predictive value of PFAS compounds for all-cause mortality in diabetic patients, we performed diagnostic value analysis using ROC curves for the three compounds (PFOS, PFOA, and MPAH). The analysis results showed that these three PFAS compounds exhibited good predictive ability for all-cause mortality in diabetic patients, with area under the curve (AUC) values of 0.689 for PFOS, 0.607 for PFOA, and 0.677 for MPAH. These findings suggest that these compounds have potential application value in predicting the risk of all-cause mortality (Supplementary Figure S1).

### 3.4. Investigating the Link Between Three PFAS Compounds and All-Cause Mortality in Diabetic Individuals

**Figure 2:**
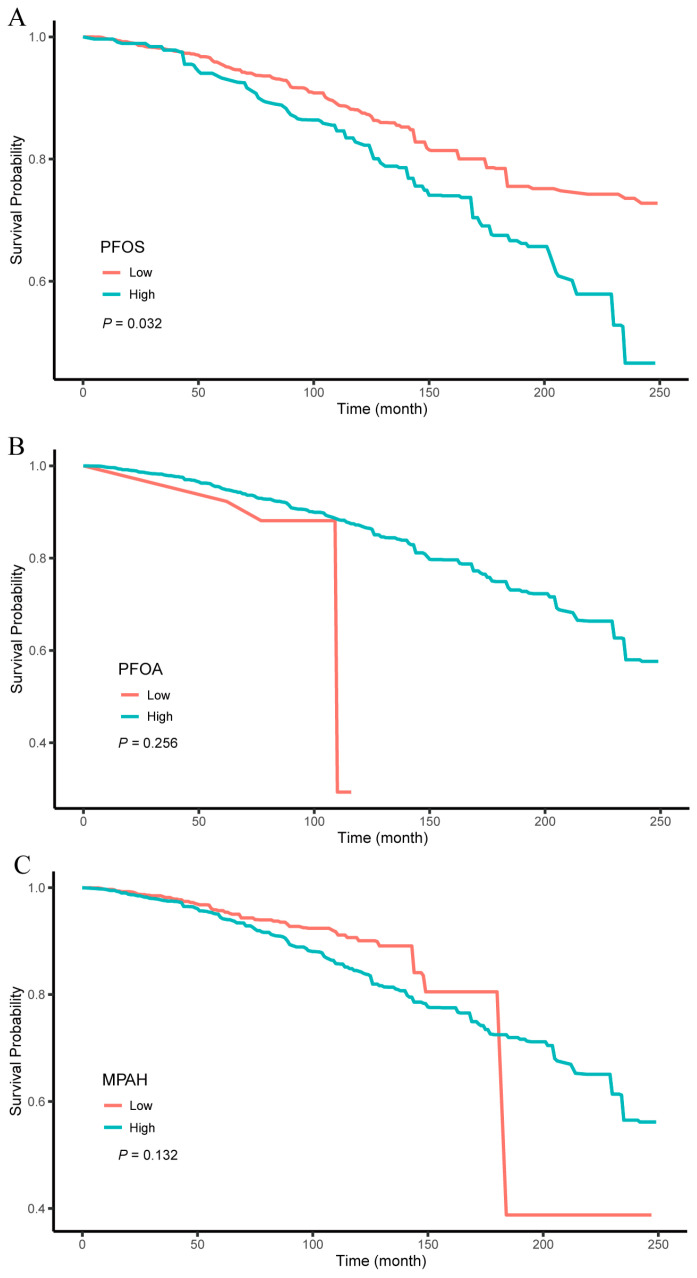
Kaplan–Meier survival curves for all-cause mortality in the diabetic population, stratified by high and low levels of PFOS (**A**), PFOA (**B**), and MPAH (**C**). The sample sizes for each group were as follows: PFOS high exposure (*n* = 195), PFOS low exposure (*n* = 1061), PFOA high exposure (*n* = 1078), PFOA low exposure (*n* = 178), MPAH high exposure (*n* = 568), MPAH low exposure (*n* = 688). Abbreviations: PFOS, perfluorooctanesulfonic acid; PFOA, perfluorooctanoic acid; MPAH, 2-(N-methyl-PFOSA) acetate acid.

We used X-tile to estimate the optimal cut-off values for PFOS, PFOA, and MPAH as 27.1, 1.1, and 0.29 ng/mL, respectively. Based on these optimal cut-off values, the participants were classified into high- and low-level groups for each compound. Kaplan–Meier (KM) survival curves were plotted unadjusted, and log-rank tests were used to compare the differences between the high- and low-level groups. Figure 2 illustrates the diabetic patients in the high-PFOS group had a significantly increased risk of all-cause mortality (*p* = 0.032), whereas no significant differences were found between high- and low-expression groups for PFOA and MPAH (*p* > 0.05). Subsequently, we performed a weighted multivariable Cox regression analysis to examine the relationship between PFOS high/low-expression groups and mortality risk. In the fully adjusted model, accounting for variables such as age, sex, BMI, PIR, education level, race, smoking, alcohol consumption, HEI-2015, hypertension, total cholesterol, and medication use, a 1-unit rise in PFOS concentration was associated with a 55% increase in all-cause mortality risk in the high-expression group (HR 1.55, 95% CI 1.06–2.29) (Table 3).

In the sensitivity analysis, when excluding participants who died within one year of follow-up (2.4%), the hazard ratio and statistical significance for the PFOS high-expression group remained consistent with the initial analysis (HR: 1.54, 95% CI: 1.05–2.27) (Supplementary Table S1). Similarly, after constructing a fully adjusted model using the confounders identified by the DAG (Supplementary Figure S2), the PFOS high-expression group was found to be consistent with the initial analysis (HR: 1.49, 95% CI: 1.02–2.17) (Supplementary Table S2), indicating the robustness of our findings. Additionally, the sensitivity analysis confirmed that the primary association results were not significantly influenced by a small number of extreme events.

### 3.5. Results of Toxicogenomic Analysis

**Figure 3:**
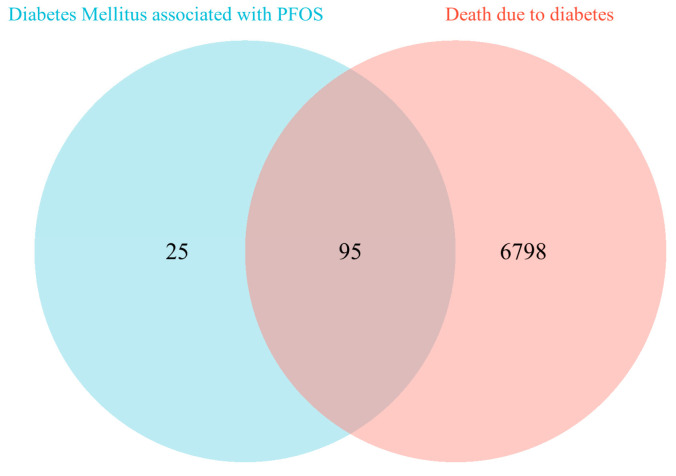
Venn diagram of PFOS-related diabetes genes (CTD) and “Death due to diabetes” genes (GeneCards). Abbreviations: PFOS, perfluorooctanesulfonic acid; CTD, Comparative Toxicogenomics Database.

Through the Comparative Toxicogenomics Database (CTD), 120 genes were identified as related to PFOS and diabetes, while the GeneCards database revealed 6893 genes associated with “death due to diabetes”. Intersection analysis yielded 95 overlapping genes (Figure 3), the details of which are listed in Supplementary Table S3.

**Figure 4:**
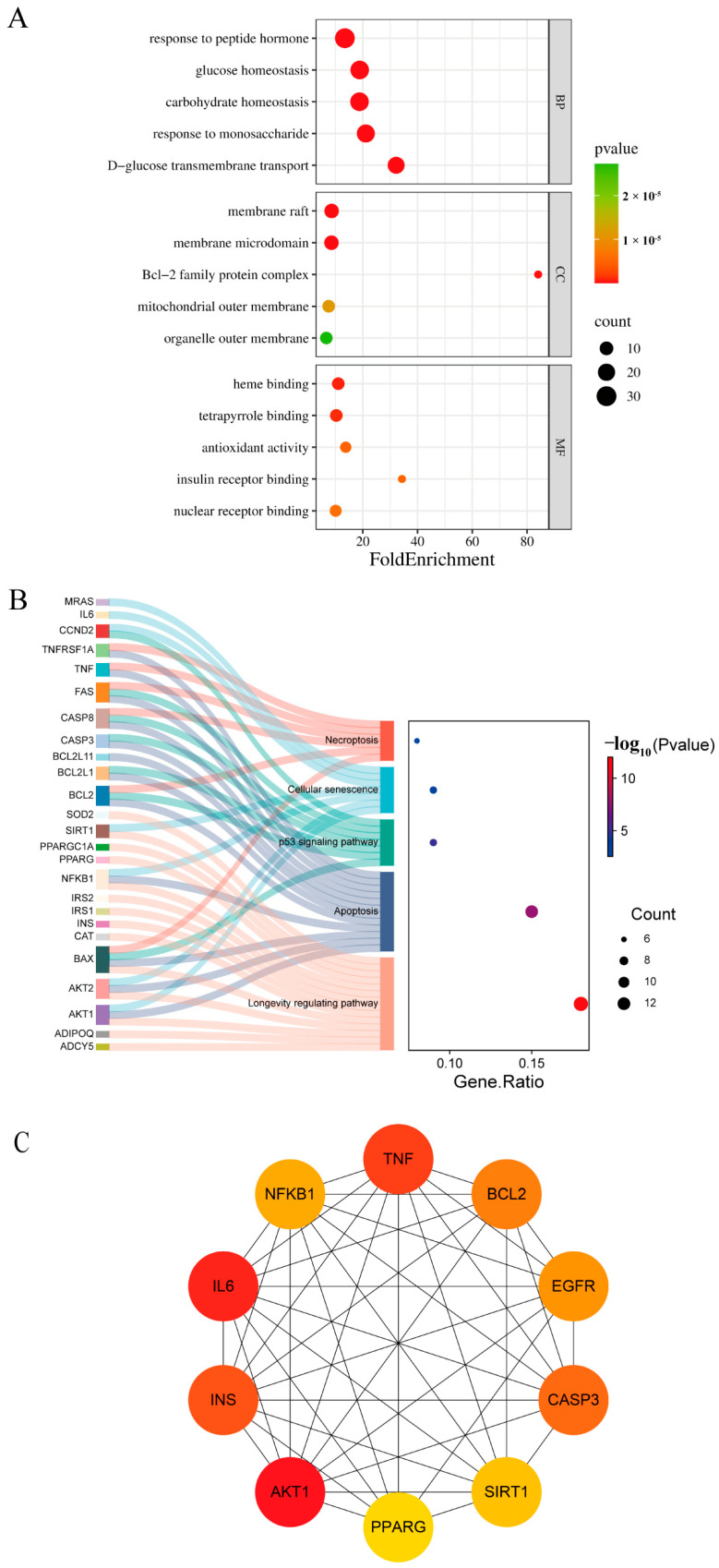
Results of the toxicogenomic analysis: (**A**) The top five biological processes (BP), cellular component (CC), and molecular functions (MF) associated with diabetes-related mortality after PFOS exposure. (**B**) KEGG pathway analysis. (**C**) Identification of 10 hub genes from the PPI subnetwork. Abbreviations: PFOS, perfluorooctanesulfonic acid; BP, biological processes; CC, cellular component; MF, molecular functions; KEGG, Kyoto Encyclopedia of Genes and Genomes; PPI, protein-protein interaction; AKT1, AKT serine/threonine kinase 1; BCL2, B-cell leukemia/lymphoma 2 apoptosis regulator; CASP3, caspase 3; IL6, interleukin 6; TNF, tumor necrosis factor; PPARG, peroxisome proliferator-activated receptor gamma; SIRT1, sirtuin 1; INS, insulin; NFKB1, nuclear factor kappa B subunit 1; EGFR, epidermal growth factor receptor.

To explore the mechanisms through which PFOS may affect mortality in diabetic patients, functional enrichment analysis of the 95 genes was performed. Figure 4A presents the top five enriched GO annotations in each category. In the biological process (BP) category, the top enriched terms included *response to peptide hormone*, *glucose homeostasis*, *carbohydrate homeostasis*, *response to monosaccharide*, and *D-glucose transmembrane transport*, with *response to peptide hormone* being the most enriched, involving 30 genes. In the cellular component (CC) category, the main enriched terms were *membrane raft*, *membrane microdomain*, *Bcl-2 family protein complex*, *mitochondrial outer membrane*, and *organelle outer membrane*. Among these, *membrane raft* was the most significant, with 12 genes participating. In the molecular function (MF) category, key enriched terms included *heme binding*, *tetrapyrrole binding*, *antioxidant activity*, *insulin receptor binding*, and *nuclear receptor binding*, with *insulin receptor binding* involving four genes. KEGG pathway analysis further highlighted key pathways related to aging and mortality (Figure 4B), such as the *longevity regulating pathway* (14 genes), *apoptosis* (12 genes), and *p53 signaling pathway*, suggesting that PFOS may influence survival through these biological pathways.

A protein–protein interaction (PPI) network was constructed to identify critical regulatory genes among the 95 candidates. The network consisted of 94 nodes and 306 edges, with highly connected nodes indicating potential biological importance. Using the Cytoscape plugin cytoHubba, the top 10 hub genes were ranked by connectivity (Figure 4C), where darker colors represented higher rankings and potentially more significant regulatory roles.

To determine the effects of PFOS on the protein activity, mRNA expression, and protein expression of the top 10 genes, this study analyzed individual chemical–gene interactions using the CTD tool. As shown in Table 4, PFOS was found to have the ability to upregulate or downregulate mRNA expression, protein activity, or protein expression. Based on the analysis, it was observed that PFOS exposure leads to a decrease in both the mRNA and protein expression of B-cell leukemia/lymphoma 2 apoptosis regulator (BCL2) while promoting the mRNA expression and protein activity of peroxisome proliferator-activated receptor gamma (PPARG). Notably, the most significant interactions were observed in relation to the mRNA expression of BCL2 and PPARG.

## 4. Discussion

In this prospective cohort study, we included 1256 adult diabetic patients and examined the serum concentrations of seven PFAS compounds (PFOS, PFOA, PFHxS, PFNA, PFUS, PFDE, and MPAH) and their association with all-cause mortality. Preliminary logistic regression analysis revealed that the concentrations of PFOS, PFOA, and MPAH were associated with all-cause mortality in diabetic patients. Further Cox proportional hazards regression analysis showed a significant association between high PFOS levels and all-cause mortality, while no significant differences were observed for PFOA and MPAH. In the toxicogenomic analysis, we identified diabetes-related genes and genes associated with diabetes-induced mortality associated with PFOS. GO and KEGG enrichment analyses suggested that PFOS may influence diabetes patient survival by regulating signaling pathways related to aging and apoptosis. Additionally, PPI network analysis identified key hub genes, highlighting the potential involvement of the PPARα signaling pathway in the prognosis of diabetes patients exposed to PFOS. The study suggests that PFOS could significantly influence mortality risk in diabetic patients, highlighting the importance of addressing PFOS exposure and its potential public health implications. To the best of our knowledge, comprehensive studies examining the potential link between PFAS compounds and all-cause mortality in diabetes are limited, and our study aimed to address this gap. Future research should further investigate the biological mechanisms of PFAS and their role in the management of diabetes to provide new insights for improving the health outcomes of diabetic patients.

Our study results indicate that higher levels of PFOS were significantly associated with elevated all-cause mortality in the diabetic cohort. This finding is indirectly supported by other studies. Increasing attention is being given to the potential health risks of PFAS, including cancer, endocrine disruption, and immune system effects [12], particularly concerning mortality. A retrospective ecological study has shown a statistical association between PFOA and PFNA levels in drinking water and the incidence of thyroid cancer in the United States [24]. A systematic review of epidemiological studies suggests that endocrine-disrupting chemicals (EDCs) such as PFAS may increase the risk of breast cancer, particularly with early-life exposure [25]. PFAS exposure has also been associated with elevated inflammatory markers (e.g., IL-6), which may affect the immune system of women during pregnancy and postpartum [26]. These studies suggest that PFAS is associated with a variety of health issues, which may indirectly impact an individual’s survival rate. In a study on the U.S. adult population, researchers found that individuals in the high-exposure group of PFAS mixtures had a significantly elevated risk of all-cause mortality compared to the low-exposure group. Specifically, in the analysis of individual PFAS components, high concentrations of PFOS (perfluorooctane sulfonic acid) exposure were associated with increased all-cause mortality as well as mortality from heart disease and cancer in U.S. adults [27]. Additionally, a study in Italy revealed an association between PFAS exposure and cardiovascular disease and cancer mortality. This study analyzed mortality data from residents living in PFAS-contaminated areas and found that these residents had significantly higher all-cause mortality than expected. Notably, mortality from heart disease and kidney cancer was significantly increased in the exposed population [28], further supporting the potential link between PFAS and mortality. Additionally, our study identified a risk threshold for serum PFOS concentration in diabetic patients at 27.1 ng/mL. This finding may provide important insights for the personalized management of diabetes in the future. It is noteworthy that while both PFOA and MPAS were associated with total mortality in diabetic patients in logistic regression, they did not show significant associations in subsequent survival analyses. This discrepancy may be due to the inclusion of time factor in the survival analysis. This result is consistent with recent studies; Wen et al. reported no significant association between PFOA exposure and mortality in U.S. adults [27]. Furthermore, research on the long-term health outcomes of MPAH is relatively limited. Most studies have focused on PFOS and PFOA, reflecting a lower research emphasis on MPAH, with insufficient data and research accumulation on its health impacts.

The mechanisms by which high levels of PFOS increase all-cause mortality in diabetes patients remain unclear, but limited evidence suggests that disturbances in lipid metabolism and inflammation may be key factors. PFAS exposure is considered a potential risk factor for metabolic syndrome, particularly in diseases such as obesity, diabetes, and dyslipidemia. A study based on the NHANES has shown complex patterns in the relationship between different PFAS types and conditions such as obesity and hyperlipidemia. For instance, PFHxS is negatively correlated with obesity but positively correlated with hyperlipidemia, indicating that different PFAS compounds may affect metabolic health through distinct mechanisms [5]. PFAS exposure has been associated with alterations in the oxidation and synthesis pathways of fatty acids in the liver, which could lead to liver fat accumulation and liver dysfunction [29]. In an animal model study, PFAS exposure was found to cause lipid accumulation in hepatocytes, accompanied by increased oxidative stress, further exacerbating liver damage [30]. PFAS levels were mainly associated with blood lipids and apolipoproteins, particularly those containing apoC-III, across subtypes of medium-density, low-density, and high-density lipoproteins, which can elevate cardiovascular risk [31]. Specifically, serum levels of PFOS, PFOA, and PFDeA (but not PFHxS) are positively correlated with lipoprotein subcomponents, apolipoproteins, and cholesterol levels in the complex fatty acid and phospholipid profiles [32]. PFOS, with its high environmental stability and ability to migrate across water, air, and soil, can accumulate in the human body [33]. Its lipophilic properties make it prone to accumulate in adipose tissue, potentially disrupting lipid metabolic pathways, including fatty acid oxidation and cholesterol synthesis [34]. The likely mechanism involves PFOS potentially influencing hepatic lipid metabolism in diabetes patients by activating peroxisome proliferator-activated receptors (PPARs), leading to complications such as fatty liver, which may impact patient survival [29]. Notably, this hypothesis aligns with the findings from our toxicogenomic analysis, further supporting its plausibility. Furthermore, PFAS may exacerbate the pathological processes of diabetes by enhancing chronic low-grade inflammation. Research has shown that PFAS exposure induces inflammatory responses in multiple organs. A study using NHANES 2005–2012 data found significant associations between several PFAS compounds and increased serum albumin and lymphocyte percentage, suggesting a link to chronic inflammation and oxidative stress [35]. PFAS exposure can enhance the secretion of pro-inflammatory cytokines (e.g., IL-10, IFN-γ, and TNF-α), thus promoting inflammation [36]. These cytokines not only have the potential to directly damage pancreatic β-cell function but may also worsen insulin resistance by interfering with insulin signaling pathways [37]. PFOS, a common PFAS compound, has been identified as a major driver of the elevated secretion of pro-inflammatory cytokines, including IL-6, TNF-α, and IL-1β [38]. Animal studies suggest that PFOS may activate the AIM2 DNA receptor pathway to induce pro-inflammatory cytokine secretion by macrophages, leading to inflammatory damage [39]. Therefore, PFAS exposure may contribute to the worsening of metabolic disturbances in diabetes patients through mechanisms affecting lipid metabolism, inflammation, and insulin signaling, ultimately influencing all-cause mortality. This toxicogenomic analysis further investigates the potential role of PFOS exposure in elevating all-cause mortality risk in diabetic patients through pathways associated with glucose metabolism, apoptosis, and cell signaling. Chemical–gene interaction pattern analysis revealed that PFOS exposure may impact the prognosis of diabetic patients by downregulating BCL2 and upregulating PPARG mRNA expression. However, the specific molecular mechanisms underlying this process remain to be fully elucidated.

This study has several strengths. It is the first to systematically evaluate the relationship between PFAS and all-cause mortality in diabetes patients, filling a significant gap in the existing literature on this important public health issue. Our study advances the understanding of the potential harms of long-term exposure to PFOS in diabetes patients, offering new directions for future research. Furthermore, based on our findings, public health policymakers should consider strengthening the regulation of PFAS to reduce population exposure, which could improve public health on a broader scale. However, our study has some limitations. First, although this study was designed as a prospective cohort study, exposure data were only collected at baseline, which limits our understanding of the dynamic relationship between exposure and outcomes. Many covariates, such as PIR and BMI, change over time and may influence the development of diabetes and mortality. Using only baseline data weakens our ability to capture their long-term impact on disease progression and PFAS exposure. Future studies should incorporate repeated measurements of these covariates to better understand their role in disease development. Although we adjusted for as many covariates as possible, unmeasured covariates cannot be ruled out. One notable factor not considered in our analysis is urban population density, which could serve as a potential confounder. Urban living is often associated with higher levels of environmental stressors, including PFAS exposure, and may also affect socioeconomic status, health behaviors, and access to healthcare. Unfortunately, the NHANES dataset does not include data on urban population density, limiting our ability to account for this factor in our analysis. Future research could explore the role of urbanization in shaping the relationship between PFAS exposure and health outcomes. Additionally, the lack of in vitro cell and animal studies means that our understanding of the mechanisms by which PFAS compounds affect diabetes patients remains limited. Fourth, the NHANES data primarily represent the U.S. population, potentially restricting the applicability of our findings to wider populations. Therefore, future clinical research should aim to expand the study population and incorporate laboratory-based studies to further elucidate the relationship between PFAS exposure and outcomes in diabetes patients.

## 5. Conclusions

This extensive national study identified a positive correlation between PFOS exposure and all-cause mortality among diabetes patients. Further toxicogenomic analysis suggests that PFOS may influence survival by regulating pathways associated with glucose metabolism, apoptosis, and cell signaling. These findings highlight the potential public health implications of PFOS exposure in the management of diabetes. Future work should further explore the mechanisms of PFAS and their potential impact on clinical decision making to improve the prognosis of diabetes patients.

## Figures and Tables

**Table 1:** Baseline characteristics of the study participants (*n* = 1256) in the NHANES 1999–2018 survey.

Characteristics	Total (N = 1256)	Alive (N = 1044)	Death (N = 212)	*p*-Value
Age, *n* (%)				<0.0001
18–59	621 (61.685)	572 (65.930)	49 (34.308)	
≥60	635 (38.315)	472 (34.070)	163 (65.692)	
Sex, *n* (%)				0.14
Male	627 (49.053)	507 (48.039)	120 (55.598)	
Female	629 (50.947)	537 (51.961)	92 (44.402)	
Race/Ethnicity, *n* (%)				0.016
White	386 (57.654)	285 (56.043)	101 (68.044)	
Black	324 (15.475)	274 (15.495)	50 (15.346)	
Other	546 (26.872)	485 (28.463)	61 (16.610)	
PIR, *n* (%)				<0.0001
<1.3	421 (24.454)	344 (23.788)	77 (28.748)	
1.3–3.5	517 (40.072)	412 (37.784)	105 (54.830)	
>3.5	318 (35.473)	288 (38.428)	30 (16.422)	
Educational attainment, *n* (%)				0.021
Below high school	235 (9.638)	177 (8.699)	58 (15.694)	
High school	507 (40.107)	415 (39.418)	92 (44.553)	
Above high school	514 (50.255)	452 (51.884)	62 (39.753)	
Smoking status, *n* (%)				0.02
Never	677 (55.467)	586 (57.263)	91 (43.886)	
Former	365 (27.707)	286 (26.853)	79 (33.212)	
Now	214 (16.826)	172 (15.884)	42 (22.901)	
Alcohol consumption, *n* (%)				<0.0001
Never	225 (15.002)	182 (14.296)	43 (19.559)	
Moderate	151 (14.672)	142 (16.275)	9 (4.335)	
Mild	393 (37.568)	347 (39.372)	46 (25.938)	
Former	302 (19.342)	211 (16.207)	91 (39.560)	
Heavy	185 (13.416)	162 (13.851)	23 (10.608)	
BMI, *n* (%)				0.02
<18	169 (11.641)	124 (10.641)	45 (18.094)	
18–25	363 (25.341)	301 (24.985)	62 (27.635)	
>25	724 (63.018)	619 (64.374)	105 (54.271)	
Hypertension, *n* (%)				0.049
No	526 (41.110)	448 (42.290)	78 (33.503)	
Yes	730 (58.890)	596 (57.710)	134 (66.497)	
Total cholesterol, (mean ± SD), mg/dl	194.183 ± 1.782	193.331 ± 1.801	199.675 ± 5.020	0.215
HEI-2015 score, (mean ± SD)	50.129 ± 0.584	50.251 ± 0.633	49.341 ± 1.131	0.464
Taking anti-diabetic medication				0.55
No	552 (42.863)	462 (43.243)	90 (40.414)	
Yes	704 (57.137)	582 (56.757)	122 (59.586)	
PFOS, median (IQR), ng/mL	11.01 (5.40, 20.53)	10.10 (5.00, 17.93)	17.95 (10.10, 32.23)	<0.0001
PFOA, median (IQR), ng/mL	2.60 (1.57, 4.20)	2.40 (1.47, 4.00)	3.30 (2.3, 5.05)	0.008
PFNA, median (IQR), ng/mL	0.91 (0.50, 1.40)	0.90 (0.50, 1.40)	0.91 (0.57, 1.48)	0.53
PFHS, median (IQR), ng/mL	1.59 (0.90, 2.60)	1.50 (0.80, 2.50)	1.90 (1.19, 3.00)	0.175
PFDE, median (IQR), ng/mL	0.20 (0.14, 0.40)	0.19 (0.14, 0.40)	0.20 (0.19, 0.33)	0.472
PFUA, median (IQR), ng/mL	0.14 (0.07, 0.20)	0.14 (0.07, 0.20)	0.18 (0.14, 0.20)	0.121
MPAH, median (IQR), ng/mL	0.20 (0.07, 0.40)	0.20 (0.07, 0.37)	0.37 (0.20, 0.63)	0.003

Abbreviations: SD, standard deviation; IQR, interquartile range, BMI, body mass index; PIR, poverty income ratio; HEI, healthy eating index; PFOS, perfluorooctanesulfonic acid; PFOA, perfluorooctanoic acid; PFNA, perfluorononanoic acid; PFHxS, perfluorohexanesulfonic acid; PFUS, perfluoroundecanoic acid; PFDE, fluorodecanoic acid; MPAH, 2-(N-methyl-PFOSA) acetate acid. The HEI-2015 score is composed of food groups and nutrient indicators that score for adequacy or moderation. PIR: calculated by dividing the family income by the poverty threshold. Alcohol consumption: “Never” < 12 drinks in lifetime, “Former” ≥ 12 drinks in 1 year and no drink last year or no drink last year but ≥12 drinks in lifetime, “Mild” < 1 drinks/d for female and <2 drinks/d for male, “Moderate” 1 to 2 drinks/d is for females and 2 to 3 drinks/d is for males, and “Heavy” ≥3 drinks/d is for females and ≥4 drinks/d is for males.

**Table 2:** Logistic regression results on the association between PFAS compounds and death in the DM population.

	Crude Model		Model 1		Model 2		Model 3	
PFAS	OR (95%CI)	*p*-Value	OR (95%CI)	*p*-Value	OR (95%CI)	*p*-Value	OR (95%CI)	*p*-Value
PFOS	1.03 (1.02, 1.04)	<0.0001	1.03 (1.01, 1.04)	<0.001	1.03 (1.01, 1.04)	<0.001	1.03 (1.01, 1.04)	<0.001
PFOA	1.09 (1.03, 1.14)	0.002	1.07 (1.00, 1.15)	0.04	1.10 (1.02, 1.18)	0.01	1.11 (1.03, 1.19)	0.01
PFNA	1.05 (0.90, 1.23)	0.52	1.03 (0.85, 1.25)	0.75	1.07 (0.90, 1.27)	0.45	1.07 (0.90, 1.26)	0.44
PFHS	1.05 (0.98, 1.12)	0.16	1.00 (0.93, 1.09)	0.92	1.00 (0.93, 1.08)	0.97	1.00 (0.93, 1.08)	0.96
PFDE	1.26 (0.72, 2.20)	0.41	1.24 (0.63, 2.42)	0.53	1.33 (0.70, 2.50)	0.38	1.23 (0.65, 2.33)	0.52
PFUA	1.60 (0.89, 2.85)	0.11	1.56 (0.83, 2.94)	0.17	1.82 (0.98, 3.38)	0.06	1.77 (0.96, 3.24)	0.07
MPAH	2.51 (1.86, 3.39)	<0.0001	2.09 (1.55, 2.83)	<0.0001	2.13 (1.50, 3.02)	<0.0001	1.97 (1.39, 2.81)	<0.001

Model 1 adjusted age, sex, race, education, and PIR. Model 2 adjusted Model 1 plus other parameters, including body mass index, smoking status (never, former, and now), drinking status (never, former, moderate, mild, and heavy), and Healthy Eating Index 2015 score. Model 3 adjusted Model 2 plus other parameters, including total cholesterol, hypertension, and taking anti-diabetic medication. Abbreviations: DM, diabetes mellitus; OR, odds ratio; CI, confidence interval; PIR, poverty income ratio; PFOS, perfluorooctanesulfonic acid; PFOA, perfluorooctanoic acid; PFNA, perfluorononanoic acid; PFHxS, perfluorohexanesulfonic acid; PFUS, perfluoroundecanoic acid; PFDE, fluorodecanoic acid; MPAH, 2-(N-methyl-PFOSA) acetate acid.

**Table 3:** Cox regression analysis on the association between PFOS and all-cause mortality in diabetic population.

Character	Se	*p*-Value	HR (95% CI) *
PFOS			
Low level	ref	ref	ref
High level	0.172	0.025	1.554 (1.056, 2.287)
Age			
18–59	ref	ref	ref
≥60	0.19	<0.0001	3.639 (2.442, 5.424)
Sex			
Male	ref	ref	ref
Female	0.171	0.051	0.672 (0.450, 1.001)
Race/Ethnicity			
White	ref	ref	ref
Black	0.223	0.225	0.795 (0.548, 1.152)
Other	0.239	0.022	0.530 (0.308, 0.912)
Education			
Below high school	ref	ref	ref
High school	0.25	0.227	0.715 (0.415, 1.232)
Above high school	0.266	0.78	1.085 (0.614, 1.916)
PIR			
<1.3	ref	ref	ref
1.3–3.5	0.19	0.935	1.016 (0.697, 1.479)
>3.5	0.268	0.011	0.467 (0.259, 0.842)
BMI			
<18	ref	ref	ref
18–25	0.242	0.005	0.512 (0.322, 0.817)
>25	0.23	0.02	0.559 (0.342, 0.914)
Smoking status			
never	ref	ref	ref
former	0.199	0.613	1.116 (0.728, 1.711)
now	0.228	0.049	1.688 (1.003, 2.840)
Alcohol consumption			
never	ref	ref	ref
moderate	0.426	0.005	0.255 (0.098, 0.663)
mild	0.254	0.078	0.576 (0.312, 1.064)
former	0.243	0.937	0.979 (0.576, 1.665)
heavy	0.335	0.223	0.619 (0.286, 1.339)
HEI-2015 score	0.006	0.014	0.984 (0.972, 0.997)
Hypertension			
No	ref	ref	ref
Yes	0.179	0.251	1.277 (0.841, 1.941)
Total cholesterol	0.002	0.56	1.001 (0.998, 1.004)
Taking anti-diabetic medication			
No	ref	ref	ref
Yes	0.166	0.267	1.200 (0.870, 1.656)

* Adjusted for age, sex, race, education, PIR, body mass index, smoking status (never, former, and now), drinking status (never, former, moderate, mild, and heavy), Healthy Eating Index 2015 score, total cholesterol, hypertension, and taking anti-diabetic medication. Abbreviations: PFOS, perfluorooctanesulfonic acid; ref, reference; BMI, body mass index; PIR, poverty income ratio; HEI, healthy eating index.

**Table 4:** PFOS effects on protein activity, mRNA expression, and protein expression of the top 10 genes based on CTD chemical–gene interaction analysis.

Chemical–Gene Interaction		AKT1	BCL2	CASP3	IL6	TNF	PPARG	SIRT1	INS	NFKB1	EGFR
PFOS	mRNA expression	-	↓↓↓↓	↑	↑	↓	↑↑↑↑↓	↓	-	↓	-
	Protein expression	-	↓↓↓	-	-	-	-	↓	↑	-	-
	Protein activity	-	-	↑↑	-	-	↑↑	-	-	-	-

“↑” represents promotion of expression, “↓” represents inhibition of expression, and the number of arrows indicates the number of studies supporting the effect. Abbreviations: PFOS, perfluorooctanesulfonic acid; CTD, Comparative Toxicogenomics Database. AKT1, AKT serine/threonine kinase 1; BCL2, B-cell leukemia/lymphoma 2 apoptosis regulator; CASP3, caspase 3; IL6, interleukin 6; TNF, tumor necrosis factor; PPARG, peroxisome proliferator-activated receptor gamma; SIRT1, sirtuin 1; INS, insulin; NFKB1, nuclear factor kappa B subunit 1; EGFR, epidermal growth factor receptor.

## Data Availability

The data presented in this study are openly available on the CDC NHANES site at https://wwwn.cdc.gov/nchs/nhanes/continuousnhanes/default.aspx?BeginYear=1999 (accessed on 1 September 2024).

## Supplementary Materials

The following supporting information can be downloaded at https://www.mdpi.com/article/10.3390/toxics13030168/s1. Figure S1: Receiver operating characteristic curve analyses for PFOS, PFOA, and MPAH of all-cause mortality in the diabetic population. Figure S2: Directed acyclic graph (DAG) of the relationship between PFOS and all-cause mortality in diabetic population.; Table S1: Sensitivity analysis of the association between PFOS and all-cause mortality after excluding individuals who died within one year.; Table S2: Sensitivity analysis of the association between PFOS and all-cause mortality after adjusting for confounders identified by the directed acyclic graphs; Table S3: Detailed list of overlapping genes between PFOS-related diabetes genes (CTD) and “Death due to diabetes” genes (GeneCards).
