# Supplementary material for: Association Between Per- and Polyfluoroalkyl Substances and All-Cause Mortality in Diabetic Patients: Insights from a National Cohort Study and Toxicogenomic Analysis
Source: Toxics. 2025 Feb 27;13(3):168. doi: 10.3390/toxics13030168 (PMC11945897; doi:10.3390/toxics13030168)
Supplement: Supplementary file 1 [file toxics-13-00168-s001.zip › toxics-3417003-supplementary.pdf]

Association Between Per- and Polyfluoroalkyl Substances and All-Cause Mortality in Diabetic Patients: Insights from a National Cohort Study and Toxicogenomic Analysis

Supplementary Materials：

Supplementary Table S1. Sensitivity analysis of the association between PFOS and all-cause mortality after excluding individuals who died within one year.

Supplementary Table S2. Sensitivity analysis of the association between PFOS and all-cause mortality after adjusting for confounders identified by the directed acyclic graphs.

Supplementary Table S3. Detailed list of overlapping genes between PFOS-related diabetes genes (CTD) and “Death due to diabetes” genes (GeneCards)

Supplementary Figure S1. Receiver operating characteristic curve analyses for PFOS, PFOA, and MPAH of all-cause mortality in the diabetic population.

Supplementary Figure S2. Directed acyclic graph (DAG) of the relationship between PFOS and all-cause mortality in diabetic population.

Supplementary Table S1. Sensitivity analysis of the association between PFOS and all-cause mortality after excluding individuals who died within one year.

Character	Se	p-Value	HR (95% CI)*
PFOS			
Low level	ref	ref	
High level	0.174	0.028	1.543 (1.049, 2.271)
Age			
18-59	ref	ref	ref
≥60	0.191	<0.0001	3.352 (2.277, 4.934)
Sex			
Male	ref	ref	ref
Female	0.172	0.044	0.661 (0.441, 0.989)
Race/Ethnicity			
white	ref	ref	ref
black	0.223	0.277	0.809 (0.553, 1.185)
other	0.245	0.014	0.500 (0.287, 0.868)
Education			
below High school	ref	ref	ref
High school	0.251	0.169	0.678 (0.391, 1.179)
above High school	0.267	0.92	1.030 (0.576, 1.842)
PIR			
< 1.3	ref	ref	ref
1.3-3.5	0.193	0.856	1.037 (0.698, 1.542)
>3.5	0.272	0.013	0.465 (0.253, 0.853)
BMI			
< 18	ref	ref	ref
18-25	0.245	0.006	0.512 (0.319, 0.822)
>25	0.234	0.016	0.543 (0.330, 0.894)
Smoking status			
never	ref	ref	ref
former	0.201	0.448	1.184 (0.765, 1.833)
now	0.233	0.056	1.691 (0.987, 2.898)
Alcohol consumption			
never	ref	ref	ref
moderate	0.428	0.005	0.252 (0.097, 0.658)
mild	0.257	0.069	0.563 (0.303, 1.045)
former	0.246	0.875	0.958 (0.562, 1.634)
heavy	0.345	0.129	0.556 (0.260, 1.186)
HEI-2015 score	0.006	0.024	0.985 (0.973, 0.998)
Hypertension			
No	ref	ref	ref
Yes	0.182	0.192	1.333 (0.866, 2.052)
Total cholesterol	0.002	0.723	1.001 (0.997, 1.004)

Take anti-diabetic medication			
No	ref	ref	ref
Yes	0.168	0.223	1.229 (0.882, 1.712)

* Adjusted for age, sex, race, education, PIR, body mass index; smoking status (never, former, now), drinking status (never, former, moderate, mild, and heavy), Healthy Eating Index 2015 score, total cholesterol, hypertension, and take anti-diabetic medication.

Abbreviations: PFOS, perfluorooctanesulfonic acid; ref, reference; BMI, body mass index; PIR, poverty income ratio; HEI, healthy eating index.

Supplementary Table S2. Sensitivity analysis of the association between PFOS and all-cause mortality after adjusting for confounders identified by the directed acyclic graphs.

Character	Se	p-Value	HR (95% CI)*
PFOS			
Low level	ref	ref	ref
High level	0.172	0.040	1.485 (1.018,2.165)
Age			
18-59	ref	ref	ref
≥60	0.176	<0.0001	3.988 (2.799,5.683)
Sex			
Male	ref	ref	ref
Female	0.161	0.040	0.676 (0.465,0.982)
Race/Ethnicity			
white	ref	ref	ref
black	0.222	0.727	0.935 (0.643,1.361)
other	0.239	0.014	0.488 (0.276,0.863)
BMI			
< 18	ref	ref	ref
18-25	0.24	0.004	0.506 (0.318,0.804)
>25	0.22	0.053	0.629 (0.393,1.007)
Education			
below High school	ref	ref	ref
High school	0.245	0.100	0.639 (0.375,1.089)
above High school	0.253	0.127	0.621 (0.337,1.145)
HEI-2015 score	0.006	0.011	0.984 (0.972,0.996)

* Adjusted for age, sex, race, education, body mass index, Healthy Eating Index 2015 score;

Abbreviations: PFOS, perfluorooctanesulfonic acid; ref, reference; BMI, body mass index; HEI, healthy eating index.

Supplementary Table S3. Detailed list of overlapping genes between PFOS-related diabetes genes (CTD) and “Death due to diabetes” genes (GeneCards)

Genes
ABCC8
ADCY5
ADIPOQ
AKT1
AKT2
ATF3
ATP2A2
AUTS2
BAX
BCL2
BCL2L1
BCL2L11
BHMT
BRAF
C3
CAPN10
CASP3
CASP8
CAT
CBS
CCND2
CD36
CDKAL1
CDO1
CISD2
CPT1A
CYBA
CYP1A2
DGKD
ECE1
EDNRA
EDNRB
EGFR
ENPP1
FAS
FGF21
GCK
GCKR
GCLC
GCLM
GPD2
GPX1
GSTM1

HBA1
HHEX
HMGA1
HMOX1
HNF1B
HNF4A
HP
HPX
ICAM1
ID1
IL6
INPPL1
INS
IRS1
IRS2
LEP
LEPR
LIPC
MAPK8IP1
MAT1A
MRAS
NFKB1
NOS2
NOS3
NUS1
PEPD
PPARA
PPARG
PPARGC1A
PRKCB
PROX1
PSMD6
RETN
S100A6
SIRT1
SLC1A2
SLC2A1
SLC2A2
SLC2A4
SMAD5
SOD1
SOD2
ST6GAL1
TCF7L2

TIMP1
TNF
TNFRSF1A
TNFRSF1B
UBE2E2
UCP2
USP48
VPS26A

Abbreviations: PFOS, perfluorooctanesulfonic acid; CTD, Comparative Toxicogenomics Database.

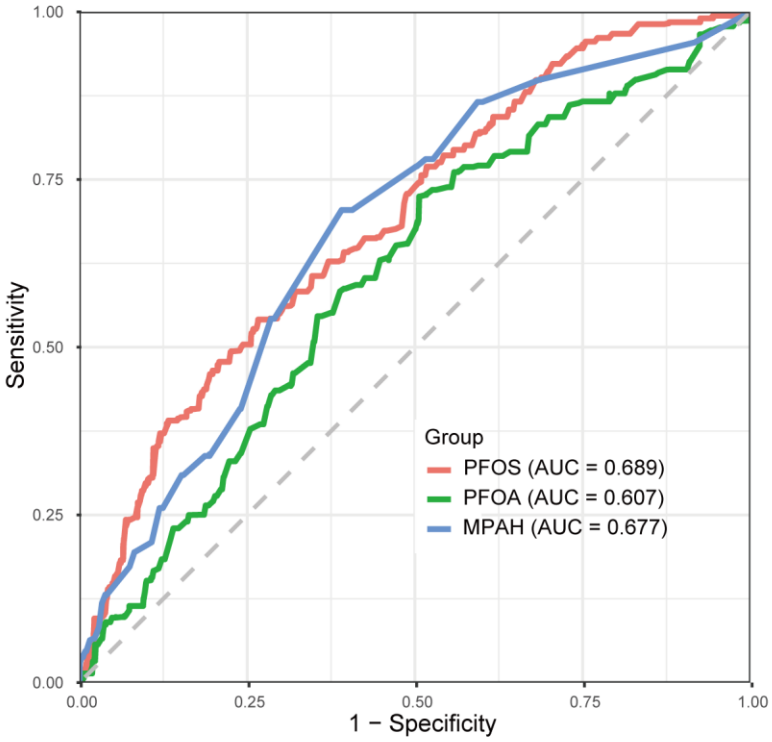

Supplementary Figure S1. Receiver operating characteristic curve analyses for PFOS, PFOA, and MPAH of all-cause mortality in the diabetic population.

Abbreviations: PFOS, perfluorooctanesulfonic acid; PFOA, perfluorooctanoic acid; MPAH, 2-(N-methyl-PFOSA) acetate acid; AUC, area under the curve.

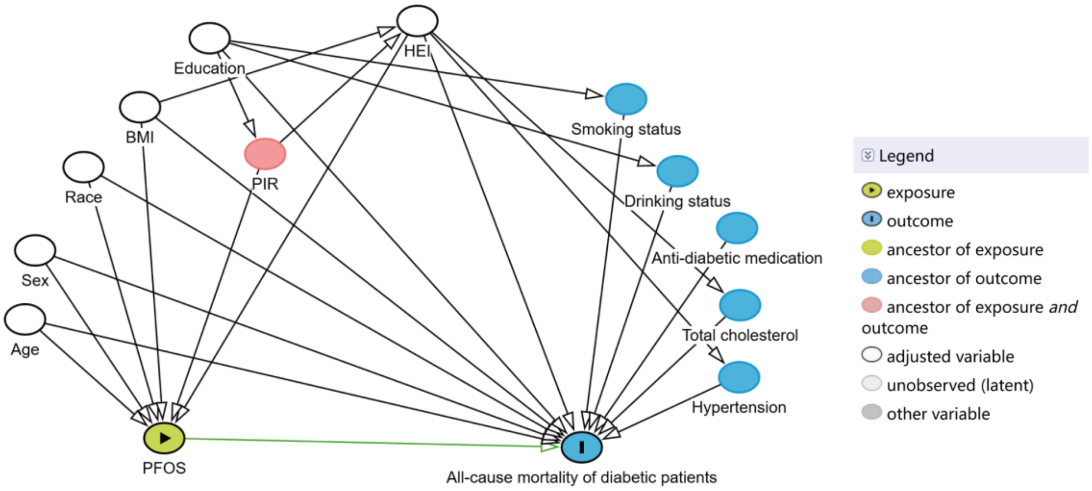

Supplementary Figure S2. Directed acyclic graph (DAG) of the relationship between PFOS and all-cause mortality in diabetic population.

Abbreviations: PFOS, perfluorooctanesulfonic acid; BMI, body mass index; PIR, poverty income ratio; HEI, healthy eating index 2015 score.
